# Thrombotic microangiopathy associated with interferon-beta treatment in patients with multiple sclerosis

**Published:** 2018-04-04

**Authors:** Seyed Mohammad Baghbanian, Abdorreza Naser Moghadasi

**Affiliations:** 1 Bualicina Hospital, Mazandaran University of Medical Sciences, Sari, Iran; 2 Multiple Sclerosis Research Center, Neuroscience Institute, Tehran University of Medical Sciences, Tehran, Iran

**Keywords:** Multiple Sclerosis, Thrombotic Microangiopathy, Interferon-Beta

Thrombotic microangiopathy (TMA) may present with acute renal failure with or without cerebral dysfunction. Pathologically, microangiopathic hemolytic anemia and thrombocytopenia lead to microvascular thrombosis occlusion and ischemia in the kidney and brain.^1^ It has been explained that there are different causes of TMA including drugs, toxins, pregnancy, infections, and autoimmunity.^2^ In the treatment of hepatitis C and induced TMA, interferon-beta (INF-β) and INF-α therapy have been reported, respectively.

It seems an inhibitory autoantibody against a disintegrin and metalloproteinase with a thrombospondin type 1 motif, member 13 (ADAMTS13) during INF-β therapy or some drugs (e.g: oral contraceptive pill, quinine) mediates ADAMTS13-acquired deficiency which leads to microvascular thrombus and platelet aggregation.^3^

We report a new TMA in a patient with multiple sclerosis (MS) who received treatment for 10 years with subcutaneous (SC) INF-β 1a. This emphasized that this risk will not decrease after a long period of time, hence clinical vigilance is necessary.

She was a 38-year-old woman with right-handed MS. The patient had not been used any other drug except INF. She started developing epistaxis and gingival hemorrhage on June 29, 2017. Her blood pressure was 190/110 mmHg, and she was afebrile. Laboratory tests are summarized in table 1. Hepatitis B surface antigen (HBS Ag) and hepatitis C virus antibody (HCV Ab) were negative. Her blood smear showed schizocytes (Figure 1). TMA was diagnosed via plasmapheresis and corticosteroid therapy. 

After 1 month, red blood cells (RBCs) became elevated to 3810/mm^3^, hemoglobin (Hgb) reached to 10.5 g/dl, and platelets increased to 65000/mm^3^, blood pressure has controlled to normal levels, and kidney has achieved normal function. 

Limited cases of TMA have been reported in patients with MS on treatment with INF-β. 

Broughton, et al. reported a late-onset TMA presented with hypertension, renal dysfunction, thrombocytopenia, and lactate dehydrogenase (LDH) elevation similar to our case. In their case, TMA was confirmed in kidney biopsy.^2^

**Table 1:** Laboratory findings in our case at admission

**Variable**	**Value**
RBC (/mm^3^)	2880
Hgb (g/dl)	6.6
Platelet (/mm^3^)	25000
BUN (mg/dl)	82
Cr (mg/dl)	1.9
LDH (mg/dl)	2561
UA	Trace proteinuria < 30 mg/dl
ALT (U/l)	25
AST (U/l)	77

RBC: Red blood cell; Hgb: Hemoglobin; BUN: Blood urea nitrogen; Cr: Creatinine; LDH: Lactate dehydrogenase; UA: Urinalysis; ALT: Alanine transaminase; AST: Aspartate transaminase

Olea, et al. reported an early-onset TMA presented with hypertension, thrombocytopenia, subnephrotic proteinuria, renal dysfunction, and elevated LDH. Kidney biopsy showed glomerular microangiopathy.^4^

**Figure 1:**
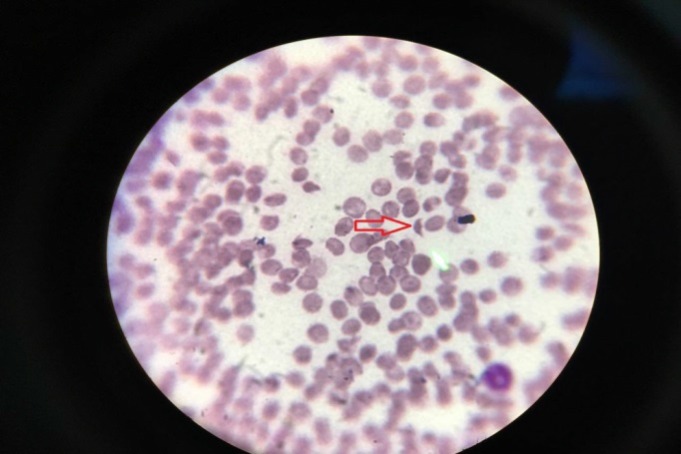
The presence of schistocytes in patient's blood smear

One other late-onset TMA presented with hypertension and renal dysfunction which was confirmed by kidney biopsy. Orvain, et al. postulated the possible role of anti-ADAMTS13 IgG antibody induced by INF-β.^5^

In Vosoughi and Marriott study, the second late-onset TMA case presented with neurological manifestation, malignant hypertension, thrombocytopenia, pulmonary edema, and generalized tonic clonic seizure, thrombocytopenia and schizocytes in the blood smear. TMA diagnosis was confirmed clinically.^6^

Our case presented in a similar manner to other late-onset TMA cases with thrombocytopenia, hypertension, and renal dysfunction. Schizocytes in blood smear and therapeutic response to the classic treatment of TMA confirmed the diagnosis. 

TMA is a rare but actually life-threatening side effect of INF-β which could present late, even after 10 years of treatment. It is our opinion healthcare providers, who monitor and follow patients with MS, are supposed to consider the early presentation of TMA, especially any elevated unexplained hypertension.

## Notes:

**How to cite this article:** Baghbanian SM, Naser Moghadasi A. Thrombotic microangiopathy associated with interferon-beta treatment in patients with multiple sclerosis. Iran J Neurol 2018; 17(2): 89-90.
